# Escape box and puzzle design as educational methods for engagement and satisfaction of medical student learners in emergency medicine: survey study

**DOI:** 10.1186/s12909-022-03585-3

**Published:** 2022-07-02

**Authors:** Christina Cantwell, Soheil Saadat, Sangeeta Sakaria, Warren Wiechmann, Gabriel Sudario

**Affiliations:** Department of Emergency Medicine, University of California, Irvine, 101 The City Drive South, RT. 128-01, Orange, CA 92868 USA

**Keywords:** Gamification, Escape box, Puzzle, Escape room, Didactics, Undergraduate medical education, Remote learning

## Abstract

**Background:**

Gamification in medical education has gained popularity over the past several years. We describe a virtual escape box in emergency medicine clerkship didactics to teach chest pain and abdominal pain and compare this instructional method to a traditional flipped classroom format.

**Methods:**

A virtual escape box was designed at our institution and incorporated into the mandatory two-week emergency medicine clerkship. The game consisted of a PDF with four cases containing puzzles to unlock a final clue. Likert scale surveys were administered to assess participants’ perceptions of the escape box format; of clerkship didactics as a whole; and of the clerkship overall. These responses were compared to the prior year’s evaluations on flipped classroom didactics and clerkship.

**Results:**

One hundred thirty-four learners participated in the escape box and completed the survey. Eighty-six percent strongly agreed with feeling more engaged with the escape box, 84% strongly agreed with learning something new, 81% strongly agreed with finding the escape box to be satisfying, 78% strongly agreed with being able to apply knowledge gained, and 74% strongly agreed with wanting more escape boxes incorporated into medical education. The escape box showed a higher average score (3.6 ± 0.63) compared to chest pain (3.5 ± 0.67) and abdominal pain (3.2 ± 0.77) flipped classroom sessions (*p* = 0.0491) for the category of “lecturer explaining content clearly and at the proper level of complexity.” For the category of “lecturer provided effective instructional materials,” the escape box showed higher scores (3.6 ± 0.63) compared to flipped classroom for chest pain (3.4 ± 0.77) and abdominal pain (3.1 ± 0.80) (*p* < 0.001).

**Conclusions:**

Escape boxes are adaptable to a virtual format and can teach abstract concepts such as teamwork and communication in addition to traditional lecture content. Ratings of didactics were higher for the escape box compared to the flipped classroom, while ratings of overall clerkship experience were not found to change significantly.

**Supplementary Information:**

The online version contains supplementary material available at 10.1186/s12909-022-03585-3.

## Background

Gamification is the incorporation of game elements into a non-game setting. In medical education, gamifying traditional lecture-based material has gained popularity as alternative methods of instruction. The rise of gamification in the medical education setting coincides with the rise of a new generation of medical learners. Guckian et al [1] coin the term “Millennial MedEd” to describe learners born between 1981–1996 who are adept at technology when examining the use of escape rooms in medical education, similar to how Bigdeli and Kaufman [2] describe the current generation of learners as “digitally native.” Bigdeli and Kaufman [2] define terminology for use of gamification in educational settings, including that of “serious games,” which serve the primary purpose of education rather than entertainment. For learners, gamified learning activities must balance learning objectives with game objectives. Game elements include the use of points, badges, or leaderboards to add competition, time limits, limited resources, and a narrative to learning objectives [3].

An escape room is a game style that provides an immersive experience for learners. Often accompanied by a storyline, escape rooms incorporate a series of puzzles (puzzle hunt) and tasks, requiring teams to complete the tasks within a time constraint to escape the room or unlock a box [4]. Escape rooms in an educational setting have been previously studied and found to have a positive effect on post-test knowledge and learner satisfaction. For example, for pharmacy students learning diabetes management through an escape room with a narrative of a patient misplacing his medication, there was a statistically significant increase in post-test knowledge after completing the session [5]. In the Thomas Jefferson Hospital system, another learner group consisting of incoming interns completed a patient safety escape room with the goal of identifying patient hazards. QR codes were incorporated as digital clues, and overall interns rated the activity as increasing engagement and was overall perceived as a successful endeavor [6]. In medical education, escape rooms have been utilized to teach processes in addition to content [1]. Additionally, with the move to virtual platforms during the COVID-19 pandemic, escape rooms provide the convenience of being adapted to a virtual setting and do not need an expert facilitator due to its intrinsic game design [1].Whereas traditionally lectures are prepared and delivered by attending physicians, an escape room generally has an accompanying manual that the facilitator uses to guide participants through the games. Therefore, an escape room can be led by a resident physician or even an advanced medical student since a step-by-step guide is provided that outlines the objectives the students must meet in order to escape and complete the lesson. Facilitators do not need to have prepared a formal lesson plan ahead of time, which can save time and expand the pool of potential facilitators.

With the transition to distance learning, the need for virtual instruction became paramount to prevent discontinuity in medical education. Here we describe a virtual escape box for an emergency medicine (EM) clerkship for third- and fourth-year medical students to teach cardiac and abdominal pathology and management. We sought to determine learners’ attitudes towards gamification in this context – including utility, engagement, satisfaction, and desire to have more game elements in medical education – and compare the virtual escape box to a traditional flipped classroom format in the prior academic year.

## Methods

### Study and game design

The study was reviewed by the Institutional Review Board and determined to be self-exempt based on institution protocol. The escape box materials were designed for compatibility with a virtual, online learning platform through a 1-h video conferencing call with a lead lecturer.

The objective of the escape box was to solve multiple puzzles within the four cases in order to decode a four-letter final clue, all within 30 min. The topics covered were chest pain and abdominal pain. Learning objectives from the case-based session were mapped to the puzzle to ensure that objectives would be met through the game process. Students were presented with learning objectives prior to starting the game when downloading the case materials. Students electronically received a PDF document containing cards for four patients. Learners were required to work together in small groups in Zoom breakout rooms to uncover passwords that were used to open QR codes to end each case. The four cases involved puzzle elements consisting of: prioritizing interventions in the correct order to reveal a permutation of letters, matching anatomic areas of an electrocardiogram to generate a three-number code, matching diagnoses to their respective radiologic findings, and summing values assigned to associated symptoms to generate another code. QR codes were used to reveal four single-letter clues that opened the escape box once all four letters were collected. At the end of the game, students left the breakout rooms and joined together in the main Zoom room. The attending lecturer led a 20-min slide-based debrief reviewing all game puzzles and relevant learning objectives related to those puzzles.

Our survey study compared the replacement of the traditional flipped classroom format with the escape box game. In previous years, students had two 1-h case-based flipped classroom learning sessions that took place in-person regarding topics in emergency medical care for patients presenting with chest pain and abdominal pain. In the flipped classroom year, learning objectives were also clearly stated on the webpage where students downloaded materials for the session. The flipped classroom format requires students to complete readings and/or watch videos on their own, prior to attending an in-person session. In an effort to meet the needs of a new generation of learners as well as comply with social distancing requirements and distance learning during the COVID-19 pandemic, a gamified version of these didactics was created as a flipped classroom substitute. Additionally, in the escape box format, chest pain and abdominal pain chief complaints were combined into one 1-h session.

### Study setting and population

This survey study took place at an academic hospital and level 1 trauma center. Our institution requires all medical students to complete a two-week clerkship in emergency medicine as a graduation requirement. Students take the two-week rotation throughout the academic year with a maximum of 4 students per rotation. Per institution policy, the clerkship provides four hours of medical student didactics to learners on the rotation over the two weeks. Our intervention takes place in the first week’s learning session.

### Measurements and key outcome measures

Three surveys were used to evaluate our escape box intervention. Students in the 2020–2021 academic year completed all three surveys. Students in the 2019–2020 academic year completed only the post-didactic and post-clerkship surveys. The post-escape box survey was completed only by students in the 2020–2021 academic year to assess students’ perceptions about the game immediately after the session was completed and in isolation from the other didactic activities offered during the two-week clerkship. This post-escape box survey was used to evaluate solely the gamified didactics, whereas the post-didactic survey and post-clerkship survey evaluate clerkship didactics as a whole and is implemented by the school of medicine as a requirement for clerkship evaluation. These post-didactic and post-clerkship surveys were already in place to evaluate didactics overall and the clerkship once completed.

#### Post-escape box survey

Students completed an anonymous, voluntary, online survey immediately after the escape box session. Questions were graded on a Likert scale of strongly disagree to strongly agree, with numeric scoring from 0 (strongly disagree) to 4 (strongly agree). Questions pertained specifically to the escape box didactics format regarding: level of engagement; ability to apply knowledge from the activity in a clinical setting; satisfaction of the escape box educational experience; learning something new through the escape box; and desire for more escape boxes to be incorporated into medical school training.

#### Post-didactic survey

At the end of the clerkship, once all didactics were completed, students received an automated email to complete an anonymous, voluntary, online survey assessing the entire didactic curriculum (including the escape box experience and case-based learning). Questions in this survey were graded on various Likert Scales from 1–4 that asked students to evaluate facilitator teaching, instructional materials, teaching effectiveness, satisfaction and relevance of the didactic session as a whole.

#### Post-clerkship survey

At the conclusion of the two-week clerkship, students were encouraged to complete an online, post-clerkship survey administered by the institution. While students were asked to evaluate all aspects of the clerkship, students were specifically asked to rate (on a 1–4 Likert scale) the quality of seminars, teaching and clerkship experience.

### Data analysis

The sample size was 134 for students who took the post-escape box survey. For the post-didactics survey, 57 students completed the survey for the escape box; 64 students completed the chest pain flipped classroom survey; and 49 students completed the abdominal pain flipped classroom survey. For the post-clerkship survey, 89 students completed the survey in 2020–2021 and 87 students completed the survey in 2019–2020. Distribution of participants’ answers are reported as absolute (N) and relative frequencies (%). Scores are reported as mean ± standard deviation (SD) and were compared between the three study groups by using analysis of variance (ANOVA). Correlation between the different questions is reported as Kendall's tau correlation coefficient (r_τ_) and p-value. Type I error was set to 0.05. SPSS Statistics version 26 (IBM SPSS Statistics for Windows, Version 26.0. Armonk, NY) was used for the analysis.

## Results

### Perceptions on escape box format

**Fig. 1:**
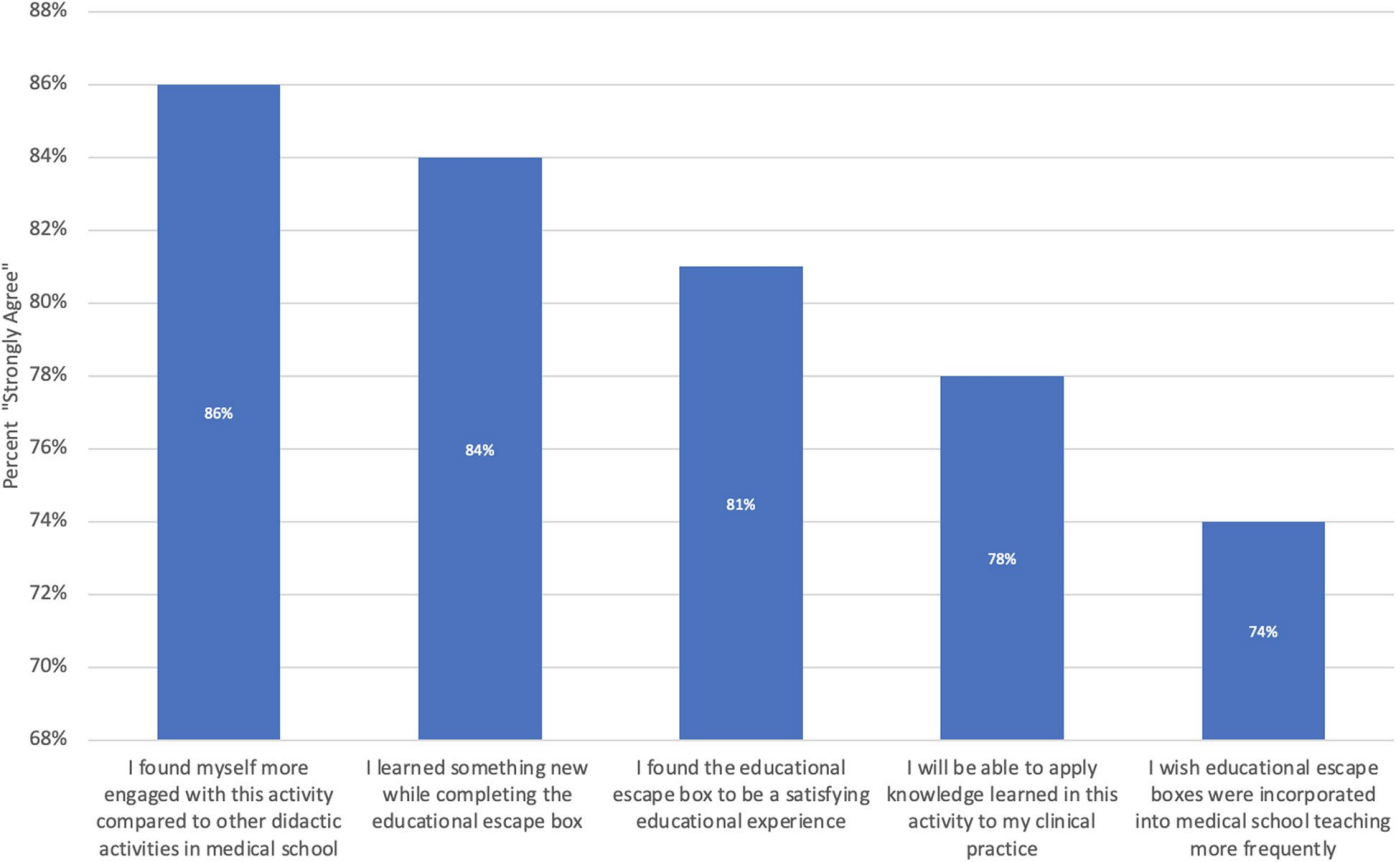
Survey on escape box didactics. Percentage of 134 survey respondents who participated in the virtual escape box from 2020–2021 and strongly agree with survey questions on engagement, learning, satisfaction, application, and desire for further escape box use

One-hundred and thirty-four learners participated in the post-escape box survey throughout the 2020–2021 academic year. Of these students, 121 were fourth-year medical students while 13 were third-year students. Among students who participated in the escape box, 86% (*n* = 115) strongly agreed with feeling more engaged with the escape box design, 84% (*n* = 112) strongly agreed with learning something new, 81% (*n* = 109) strongly agreed with finding the escape box to be a satisfying experience, 78% (*n* = 104) strongly agreed with being able to apply knowledge gained from the experience, and 74% (*n* = 99) strongly agreed with wanting to incorporate more escape boxes in medical education (Fig. 1).

Learners who ultimately indicated they strongly agreed with the statement “I wish educational escape boxes were incorporated into medical school teaching more frequently” tended to also strongly agree with the other categories (*p* < 0.001). The highest correlation was seen with finding escape boxes to be a satisfying activity (r_τ_ = 0.750, *p* < 0.001) (Table 1).

**Table 1:** Correlation between survey categories in respondents strongly agreeing with more incorporation of escape boxes in medical school

		I found myself more engaged with this activity compared to other didactic activities in medical school	I will be able to apply knowledge learned in this activity to my clinical practice	I found the educational escape box a satisfying educational experience	I learned something new while completing the educational escape box
I wish educational escape boxes were incorporated into medical school more frequently	Kendall's tau b correlation coefficient	0.464	0.543	0.750	0.637
	*P*-value	< 0.001	< 0.001	< 0.001	< 0.001
	N	134	134	134	134

In comparing third-year to fourth-year respondents, slightly more third-year students strongly agreed they learned something new from the escape box, however not to a statistically significant degree.

### Comparison in didactics evaluations between escape box and flipped classroom

We next compared differences between lecture evaluations from the 2019–2020 academic year, during which a flipped classroom model was used to teach chest pain and abdominal pain in separate sessions, and the evaluations from the 2020–2021 year, during which the flipped classrooms were replaced with a single escape box session incorporating both topics. The same group of lecturers led the sessions in both the 2019–2020 and 2020–2021 academic year. Direct comparison of a single lecturer between academic years was not feasible due to the year-round nature of the clerkship and availability of the lecturers. Fifty-seven students completed the post-didactics survey for the escape box, 64 students completed the survey for the chest pain flipped classroom, and 49 students completed the survey for the abdominal pain flipped classroom. The mean scores and standard deviations are shown in Table 2. For the category of lecturer explaining content clearly and at the proper level of complexity, the escape box showed a higher average score across raters (3.6 ± 0.63) compared to the chest pain (3.5 ± 0.67) and abdominal pain (3.2 ± 0.77) flipped classroom sessions (*p* = 0.0491). For the category of “lecturer provided effective instructional materials,” the escape box also showed higher scores (3.6 ± 0.63) compared to chest pain (3.4 ± 0.77) and abdominal pain (3.1 ± 0.80) (*p* < 0.001).

**Table 2:** Comparison of escape box to flipped classroom. Mean ± SD

Cohort	N	The lecturer explained content clearly at the proper level of complexity	The lecturer provided effective instructional materials (e.g. handouts, slides)	Effectiveness and value of teaching compared with other sessions	Applicable to my life as a physician?	Level of expertise demonstrated by the teacher	On a scale of 1–10, 10 is the most enjoyable learning experience you have encountered, how would you rate the enjoyment of this session?
Escape box	57	3.56 ± 0.627	3.632 ± 0.627	3.93 ± 0.923	3.877 ± 0.569	3.825 ± 0.63	8.772 ± 1.254
Flipped Classroom—Chest Pain	64	3.48 ± 0.666	3.375 ± 0.766	3.734 ± 0.859	4.00 ± 0.690	3.813 ± 0.774	8.281 ± 1.431
Flipped Classroom—Abdominal Pain	49	3.24 ± 0.77	3.082 ± 0.804	3.531 ± 1.09	3.939 ± 0.935	3.714 ± 0.857	8.122 ± 1.848

## Discussion

The escape box format for teaching chest pain and abdominal pain were predominantly well-received, with the vast majority of learners strongly agreeing with survey questions on engagement, learning, satisfaction, application, and desire for further escape box use. Learners who strongly agreed with incorporating escape boxes in the medical education setting also were most likely to strongly agree with reporting satisfaction as a learner. When stratified by medical school training level (i.e., third- or fourth-year medical student), fourth-year students tended to rate knowledge application, satisfaction, and desire for more escape boxes more highly than third-year students, though not to a significant degree.

In comparing students’ perceptions of didactics quality between escape box and flipped classroom, there were significant increases in rater approval in the escape box group for explaining content clearly and at the appropriate level of complexity, as well as in the category of providing effective lecture materials. While the escape box cohort learned abdominal pain and chest pain during a combined 1-h session compared to the flipped classroom cohort, which dedicated an hour to each topic separately, learners had more favorable perceptions of the escape box format in rating clear explanation of content. A possible explanation for this finding could be higher learner engagement in a game setting and more interaction with the facilitator in the discussion portion. There were also clear tasks for each puzzle within the escape box session. On the other hand, in the flipped classroom model, students were presented with a case stem in the form of a paragraph of text and open-ended questions were posed to the group. Although less time was spent in the escape box didactics sessions compared to flipped classroom, learners appear to be more receptive to the material when presented in a game style.

The ratings for the emergency medicine clerkship overall did not differ between academic years to a significant degree in categories relating to quality of conferences, experience, and teaching in the clerkship.

### Comparison with prior work

Tomaselli et al [7] reported introducing a “Price is Right” game to EM residents to teach about healthcare costs and found that 89% of participants indicated they would recommend the session to other learners, similar to our findings. We also found that learners more highly rated effectiveness of learning materials provided in the escape box format compared to the traditional flipped classroom format. Tomaselli et al [7] also reported learner preference for gamified formats in their study.

In a gamification study by Gerard et al [8], medical students, residents, and attendings were tested via a PediatricSim serious game. Game scores in their study corresponded to level of training. Further studies with our escape box lecture would be needed with a larger group to determine if level of training truly affects perception of these elements. One possibility for this trend is that students at more advanced levels of training may find the activity easier to utilize more of the clinical knowledge developed by exposure on the wards.

Deci and Ryan [9] first described the elements of competence, autonomy, and relatedness in the self-determination theory of motivation. Competence describes using challenging goals to promote higher performance; in our virtual escape box, students could demonstrate competence through understanding the pathophysiology underlying the given cases to solve the puzzles. With player autonomy, the game should allow multiple pathways to achieve a target goal; learners therefore have the element of choice in pursuing the subgoals they wish to target. Within our escape box, students were able to choose the order in which they solved cases. Relatedness is met by connecting learners with other learners or facilitators. Rutledge et al [3] apply self-determination theory to gamification in an educational setting; learners can collaborate or compete with each other, while comparing learners to other peers also stimulates motivation. Within our game, students worked together as one team to solve the cases, but future implementation of this activity could have two teams compete against each other for escaping in the shortest amount of time. The use of game elements in medical education therefore becomes very nuanced in engaging learners without distracting from the primary purpose of teaching pathophysiology and management of diseases. Furthermore, Gerard et al [8] reported in the PediatricSim game that written test scores on subject matter correlated with game scores. Therefore, use of a scoring system within the game may be a positive indicator of knowledge acquisition.

Use of game elements in medical education can also serve as a means to teach processes and more abstract lessons of communication and teamwork. Guckian et al [1] examined three examples of escape rooms in medical education, with one involving medical trainees and members of interdisciplinary fields. The learners reported enhanced communication and increased morale during the game. In another case with an obstetrics escape room, the activity was structured in such a way that every participant would need to communicate with each other [1]. Additionally, in Stanford’s Ultrasound Games, Lobo et al [10] reported a majority of participants (EM interns) strongly agreed that working in a team was an enjoyable experience and that competition helped them feel better acquainted with their peers. Escape rooms provide a method of instruction that can force communication between members or other critical action tasks as part of the game design with beneficial effects beyond didactic material. By incorporating collaboration through gamification in an otherwise mandatory training, learners can have added benefits of abstract lessons such as interprofessional and team communication or multitasking, which are competencies outlined by the Accreditation Council for Graduate Medical Education [11] emergency medicine milestones expected of EM residents.

### Limitations

Since the 2020–2021 academic year was the first year in which the escape box was utilized, further studies would be necessary to increase sample size. Additionally, pre- and post-testing could be utilized to assess learners’ knowledge acquisition from the activity. There is also a temporal element that is difficult to control for, in that students participate in the emergency medicine rotation at different times of the year based on scheduling. As students progress through training, they will naturally acquire more knowledge, which can artificially inflate scores later in the year. Similarly, the data included in our study contained evaluations from third- and fourth-year students, and the differing training levels may be confounding variables. Additionally, lecturer availability varies throughout the year, so the variety of lecturers may also be a confounding variable in having different teaching styles; however, to mitigate this effect, the group of lecturers who delivered content for the flipped classroom and escape box was consistent between the two years.

## Conclusions

The escape box format is well-received by learners and shows improved ratings in appropriate level of complexity and effective learning materials when compared to a traditional flipped classroom format. Gamification in medical education can provide a means to teach both concrete topics, such as disease diagnosis and treatment, as well as abstract skills, such as communication and teamwork. Further studies with pre- and post-game testing are needed to assess knowledge acquisition using this format of instruction in EM clerkship didactics.

## Supplementary Information

**Additional file 1.** EM Clerkship Escape Box.

**Additional file 2.**Escape Box Learning Objectives.

**Additional file 3.** Data set.

## Data Availability

All data generated or analysed during this study are included in this published article as a supplementary file.
