# Supplementary material for: Escape box and puzzle design as educational methods for engagement and satisfaction of medical student learners in emergency medicine: survey study
Source: BMC Med Educ. 2022 Jul 2;22:518. doi: 10.1186/s12909-022-03585-3 (PMC9250221; doi:10.1186/s12909-022-03585-3)
Supplement: Supplementary file 1 — Additional file 1. EM Clerkship Escape Box. [file 12909_2022_3585_MOESM1_ESM.pdf]

EM Clerkship Escape Box

It is your first day as an intern in the ED. We usually expect interns to come to four patient dispositions before they can clock out and go home! Good luck!

Helpful Hints:

- This envelope/document contains various pages that are full of clues and are interconnected.
- QR codes/links have passwords and it is your job to figure them out! All passwords are lowercase.
- If you are playing on a computer, all QR codes are clickable links.
- There is no inherent order to go through these pages. Review all of the pages before diving deep into one puzzle!

Mr. Lannister

65 y/o man presenting to the ED with chest pain. Reports he started to have L sided chest pressure while mowing the lawn 1 hour prior to arrival. He vomited twice at home and was found diaphoretic, sitting in a chair by his daughter.

He has a history of HTN, HLD, DM.

Meds: Atorvastatin, Metformin, Lisinopril

VS: 90/50, 80, 98%, 36.8

Gen: Diaphoretic, uncomfortable
Lungs: CTAB
CV: RRR no MRG
Pulses: 2+ Radial pulses

Orders:
Name: Jamie Lannister

Draw the path of interventions ordered by priority for Mr. Lannister. Note not all interventions are indicated. (No diagonal movements)

START HERE	a<br>Nitroglycerin	b<br>Esmolol
c<br>Activate cath. lab	d<br>4mg Dilaudid	END HERE
e<br>ASA	f<br>Heparin	g<br>Right sided EKG

Sign Orders Here

Davos: EKG Technician

“Crap! I mixed up the two chest pain patient medical record numbers….

I can’t remember which EKG goes with who!

I will need to log back into the EKG machine to figure this out.

The EKG machine requires a PIN number. I wrote it down in my notes in my training notebook”

Davos’ Notebook

EKG Anatomic Areas

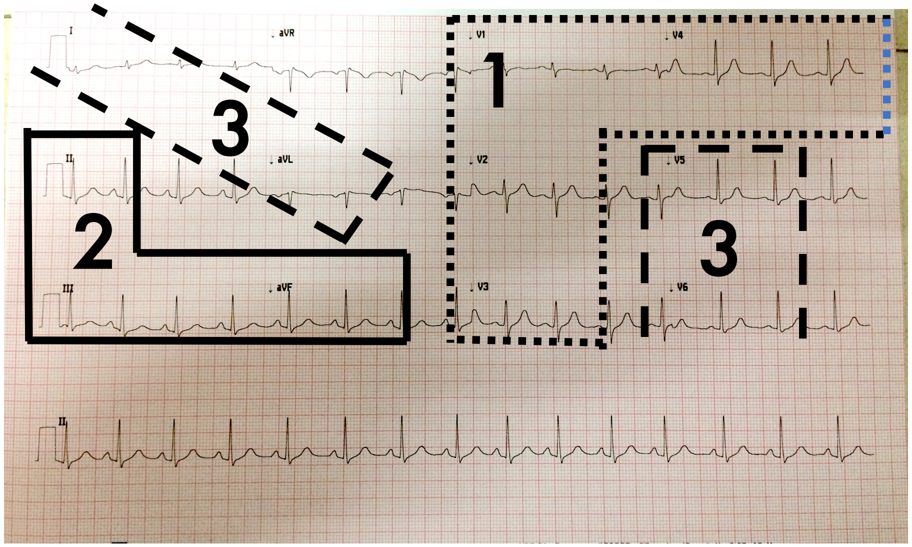

EKG Machine Access Code:

__ Lateral
__ Inferior
__ Anterior/Septal

EKG
MRN: 90210

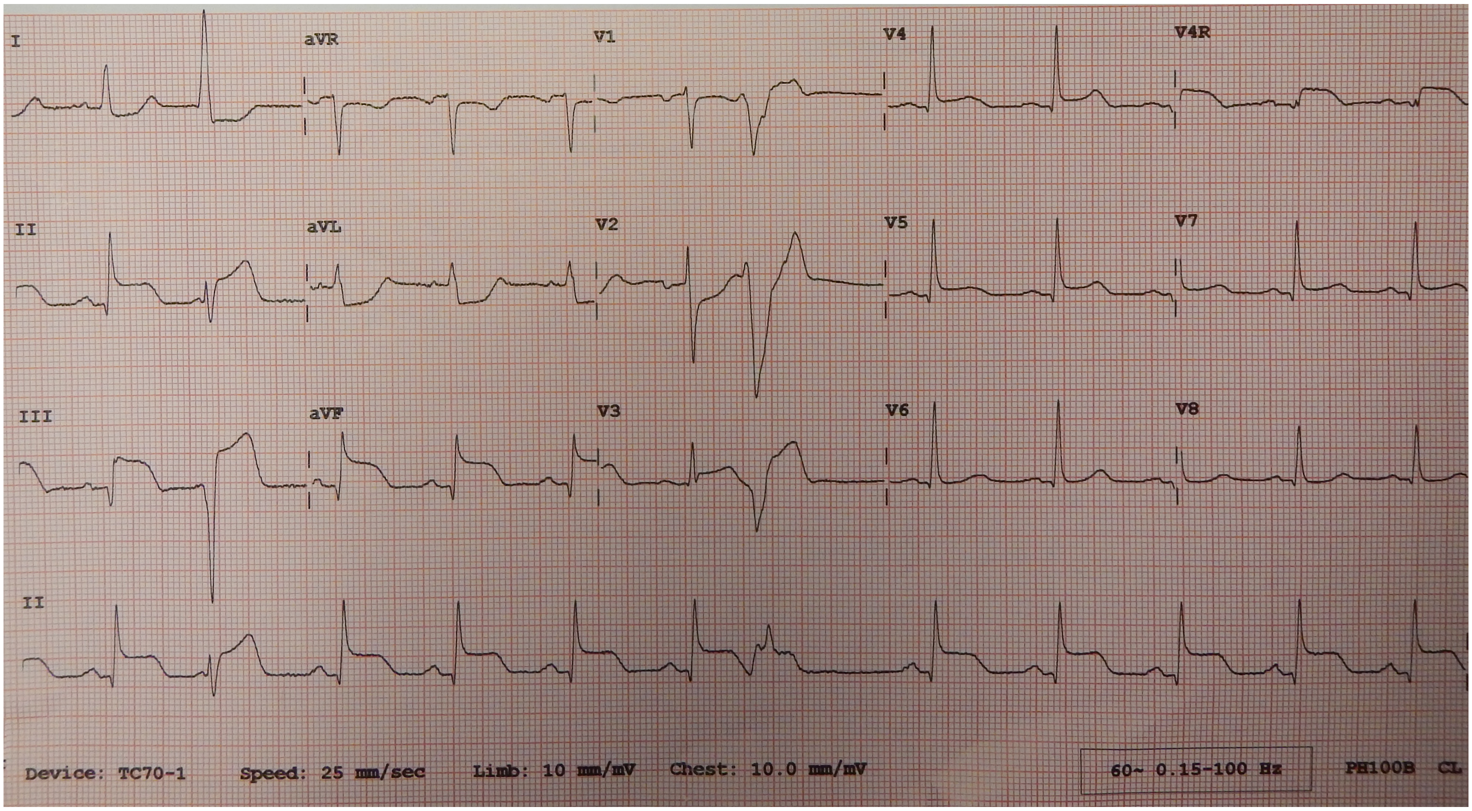

EKG
MRN: 92868

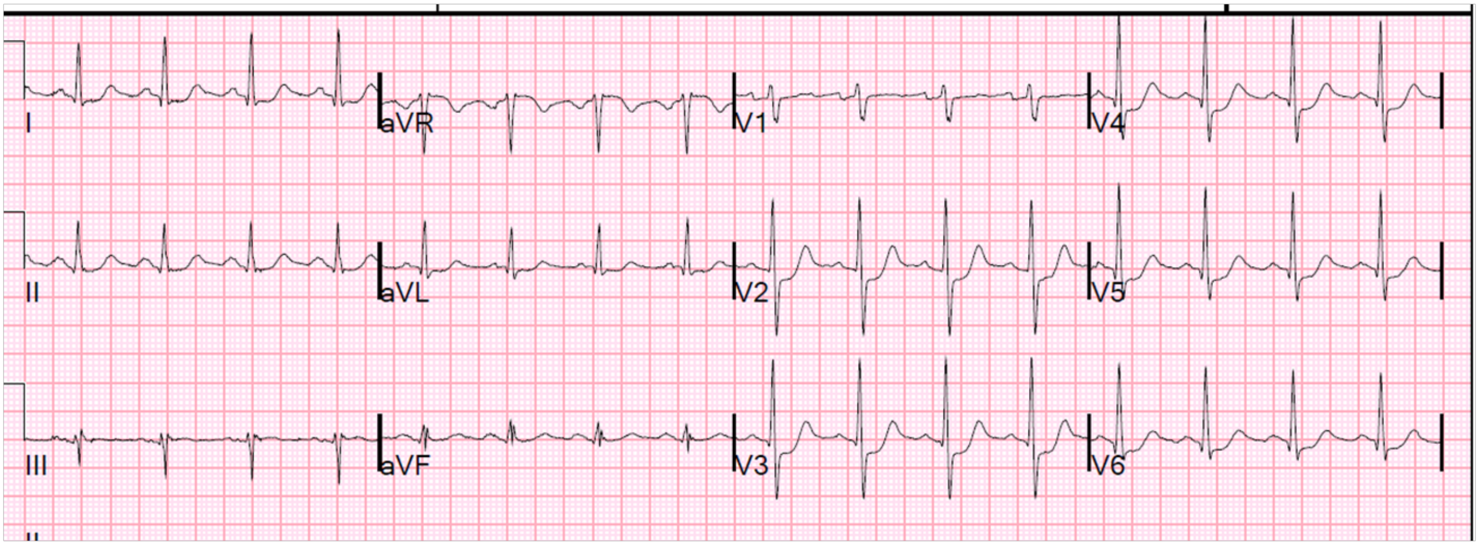

Mr. Baelish

68 y/o man presenting to the ED with nausea and vomiting. He states that he started to have nausea, sweating and chest discomfort that started after walking up 2 flights of stairs at work.

He has a history of MI in the past without intervention.

Meds: Atorvastatin, Losartan

VS: 110/70, 78, 99%, 36.8

Gen: Vomiting
Lungs: CTAB
CV: RRR no MRG
Pulses: 2+ Radial pulses

EKG Machine

Enter Access PIN Here

Cardiology Attending

Text Page Here

EHR Message

Jon Snow, RN
1:14 PM

Hi Doctor. Ms. Tarth has no orders yet. Do you mind putting some in the computer so we can start her workup?

Sign orders here

Heparin Pump

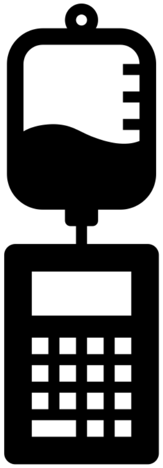

Enter AHA recommended heparin bolus for NSTEMI in units

Admit Order: Brienne Tarth

To sum it all up enter the ICD Code here to submit admission order

Clock Out

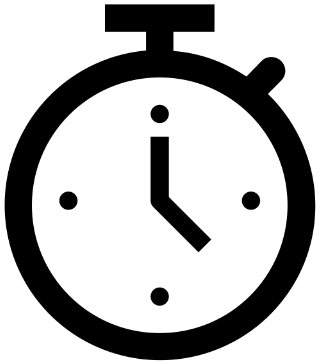

Enter Code Here

Qyburn: Pharmacist

"I see that the resident ordered heparin for Mr. Baelish.

I wonder if the resident knows that the bolus is a weight-based dose.

I'll ask the pharm resident to do a chart review to find the weight. "

Pharmacy EHR

Password hint:
Mr. Baelish EKG shows ST depressions in the ________ leads which could signify a posterior MI.

Access EHR for Chart Review Here

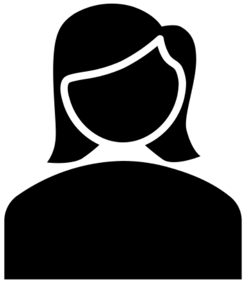

Ms. Tarth

37F presenting with abdominal pain. She is writhing in bed and says she can't answer your questions until you order her pain medications.

VS: 115, 138/69, 98% on RA, 37.7
Exam:
Gen: Appears uncomfortable, vomiting
CV: Tachycardic without murmurs
Resp: CTAB
Abd: Diffuse tenderness.

ICD Code Ms. Tarth

To admit Ms. Tarth, she will need an ICD Code. Calculate her code by identifying items associated to her diagnosis.

909 Murphy's Sign	714 Grey Turner Sign	213 Tender at McBurney's Point
949 🦂	619 Obturator Sign	310 ⬆️ Triglycerides
805 Gallstones most common cause	424 ⬆️ Total Bilirubin	760 Gallbladder wall thickening

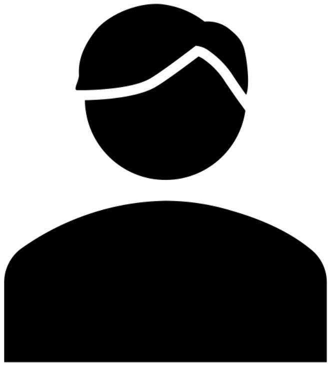

Mr. Tarly

18 year old male presenting with lower abdominal pain that started 2 days ago, worsening today. Associated nausea, vomiting and subjective fevers.

VS: 118, 140/88, 100% on RA, 38.0

Exam:
Gen: Appears uncomfortable, vomiting
CV: Tachycardic without murmurs
Resp: CTAB
Abd: Diffuse tenderness worse in the lower quadrants

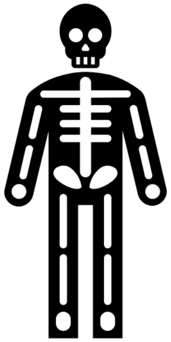

Radiologist

"It has been such a busy night! My reading queue is is getting longer and I can't catch up.

All I need to do is connect the dots to find the diagnosis.

Now, who is next on my list… Mr. Tarly."

Input Diagnosis Here

Radiology Queue

SBO ○

Cholecystitis ○

Appendicitis ○

Perforation ○

AAA ○

C

L

J

A

E

Y

S

P

T

A

P

N

R

○ 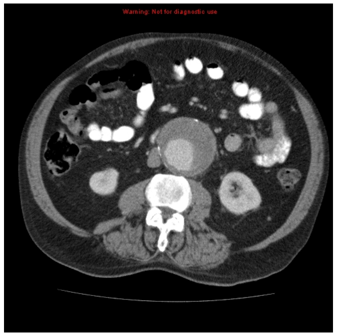

○ 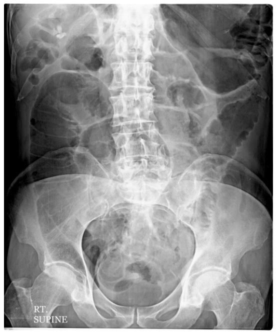

○ 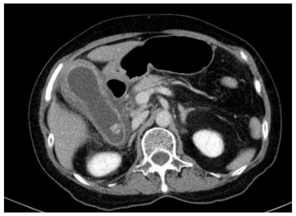

○ 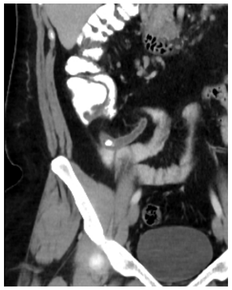

○ 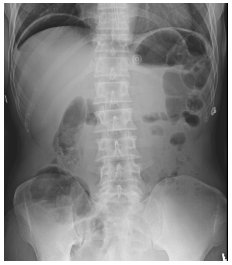

Image Source: Radiopaedia
