# Supplementary material for: Escape box and puzzle design as educational methods for engagement and satisfaction of medical student learners in emergency medicine: survey study
Source: BMC Med Educ. 2022 Jul 2;22:518. doi: 10.1186/s12909-022-03585-3 (PMC9250221; doi:10.1186/s12909-022-03585-3)
Supplement: Supplementary file 2 — Additional file 2.Escape Box Learning Objectives. [file 12909_2022_3585_MOESM2_ESM.docx]

Additional file 2: Escape Box Learning Objectives

At the end of this session, learners will be able to:

1. Identify a STEMI on ECG.
2. Localize cardiac ischemia on an ECG.
3. Summarize the emergency department treatment for inferior STEMI.
4. Calculate the appropriate heparin dosing for a patient experiencing and NSTEMI.
5. Identify 4 risk factors for acute pancreatitis.
6. Recognize the following diagnoses to corresponding abdominal imaging: cholecystitis, small bowl obstruction, hollow viscous perforation, appendicitis, abdominal aortic aneurysm
